# The value of sex-specific abdominal visceral fat as measured via CT as a predictor of clear renal cell carcinoma T stage

**DOI:** 10.1080/21623945.2021.1924957

**Published:** 2021-05-20

**Authors:** Hao Guo, Wenlei Zhao, Aijie Wang, Mingzhuo Li, Heng Ma, Fang Wang, Qing Wang, Xinru Ba

**Affiliations:** a Department of Radiology, Qilu Hospital, Cheeloo College of Medicine, Shandong University, Jinan, China; b Department of Radiology, Yuhuangding Hospital, Qingdao University School of Medicine, Yantai, China; c Department of Radiology, Yaitai Shan Hospital, Yantai, China; d Center for Big Data Research in Health and Medicine, The First Affilicated Hospital of Shandong First Medical University, Jinan, China

**Keywords:** Clear renal cell carcinoma, abdominal, obesity, computed tomography, T stage

## Abstract

Although much is known about how adipose tissue affects the development of clear cell renal carcinoma (ccRCC), little information is available for the utility of sex-specific abdominal visceral fat composition as a predictor of clear cell renal carcinoma (ccRCC) T stage. We conducted CT-based sex-specific abdominal fat measurements in ccRCC patients to assess whether VFA distribution could predict the ccRCC T stage. In total, 253 patients (182 males and 71 females) from our hospital with pathologically confirmed ccRCC (178 low T-stage and 75 high T-stage) were retrospectively reviewed for the present study. Computed tomography (CT) scans were assessed using ImageJ to differentiate between the visceral and subcutaneous fat areas (VFA and SFA), after which the relative VFA (rVFA) and total fat area (TFA) were computed. The relationships between these fat area-related variables, patient age, sex, and BMI, and ccRCC T stage were then evaluated through univariate and multivariate logistic regression analysis to clarify the association between general or sex-specific abdominal visceral fat and T stage. Following adjustment for age, males with high T stage ccRCC exhibited an increased rVFA as compared to males with low T stage ccRCC, with the same relationship being observed among females. This association between rVFA and high T stage was confirmed through both univariate and multivariate models. As thus, sex-specific visceral fat composition is a reliable independent predictor that can identify both male and female patients with high T stage ccRCC.

## Introduction

Obesity rates have risen rapidly among both males and females over the past four decades [1]. Given that obesity is closely associated with the development of a range of malignancies including renal cell carcinoma (RCC), oesophageal cancer, and liver cancer, it represents a major global health concern [1]. Indeed, obesity is thought to be second only to smoking as the most common risk factor associated with carcinogenesis [2]. While body mass index (BMI) and waist circumference values are traditionally used to measure obesity [3,4], these variables fail to accurately account for human adipose tissue distribution patterns [5]. Such fat distribution is believed to be more important than simple fat content as a predictor of obesity-related outcomes [6]. There is also direct evidence that obesity can influence the onset and progression of RCC [7], which is the deadliest type of cancer affecting the urinary system [8], and which accounts for 4% of the emerging global malignant tumour burden [9]. Visceral fat volume can more reliably predict RCC incidence and prognosis than BMI [10,11], as Kaneko et al. found that local RCC patient survival following radical resection was highly correlated with visceral fat volume, but not BMI [10]. An analysis of 116 patients with metastatic RCC undergoing antiangiogenic drug treatment similarly found that overall and progression-free survival (OS and PFS) durations were positively correlated with patient visceral and subcutaneous fat area (VFA and SFA) [11]. Sex hormones influence patterns of adipose tissue accumulation and function, with men accumulating more visceral fat on average, while in women the accumulation of both visceral and subcutaneous fat can vary with hormone levels and age [12]. It is thus important that gender be taken into account when researching the relationship between visceral fat and patient outcomes. Hu et al. recently determined that relative VFA (rVFA) can be evaluated to predict a higher Fuhrman nuclear grade in females, but not males [13]. As such, sex-specific abdominal visceral fat is likely to be associated with RCC patient prognosis. Computed tomography (CT) images are the standard approach to assess body composition in cancer patients [14], and they can be readily used to assess visceral fat distribution patterns [15]. Tumour staging is generally used to guide RCC patient prognostic evaluation and treatment [16], and as such, we conducted CT-based sex-specific abdominal fat measurements in ccRCC patients as a means of exploring whether VFA distribution could predict ccRCC T stage that has not been reported in previous studies.

## Materials and methods

### Patients and methods

Patient data from January 2014 – January 2020 was obtained from the Picture Archiving and Communication System (PACS) of Yantai Yuhuangding hospital (China). The institutional review of our hospital approved this study. The requirement for informed patient consent was waived for this retrospective study. Study inclusion criteria were as follows: (1) ccRCC patients with pathologically confirmed T stage; (2) patients with available preoperative CT images free of artefacts. Exclusion criteria were as follows: (1) ccRCC patients without pathologically confirmed T stage; (2) patients with CT image containing respiratory or other artefacts; (3) patients with other primary or secondary renal tumours; (4) patients with metabolic disorders including both Type 1 diabetes mellitus (T1DM) and Type 2 diabetes mellitus (T2DM), hypoglycaemia, thyroid dysfunction, or renal dysfunction; (5) patients that had undergone significant recent weight changes. Based upon these criteria, 253 patients (182 males and 71 females) were identified for inclusion in this study. Relevant clinical variables for these patients were analysed, including age, sex, and BMI. Patients were stratified into two groups based upon whether they had low-T-stage disease (T1 and T2) or high-T-stage disease (T3 and T4), as in prior studies [17], with sex additionally being used to categorize patients in appropriate analyses of sex.

### Image selection and abdominal fat measurement

**Figure 1.**
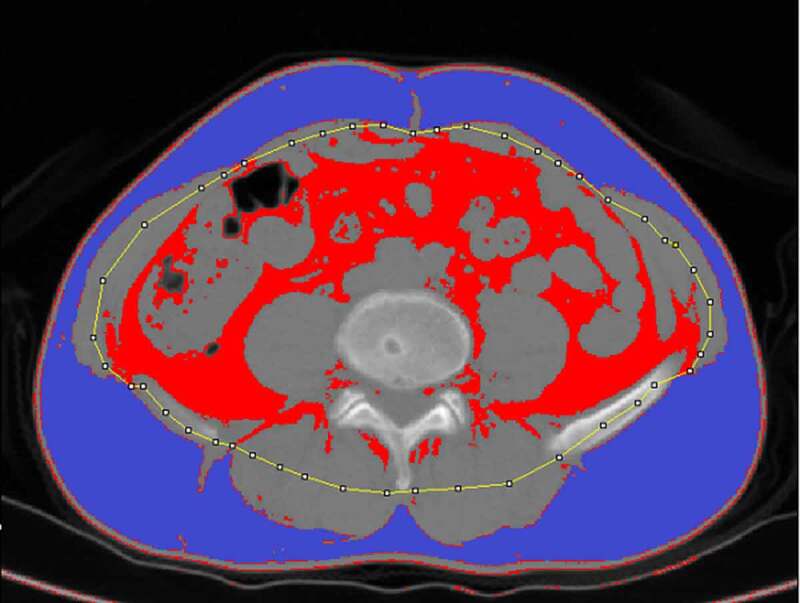
Fat measurements on CT. Subcutaneous fat (SFA, purple area) and Visceral fat (VFA, red area) were segmented after outlines (yellow line) were placed in the abdominal skeletal muscle from the original CT image for automatic calculation

Abdominal fat was separated into visceral and subcutaneous fat. Measurements were conducted as in prior reports using ImageJ 1.51 (https://imagej.nih.gov/ij/) [10,18,19], based upon preoperative CT axial images (5 mm thick) at the umbilical level (Figure 1). Rollins et al. previously reported that the total fat contents measured in the arterial and portal phase were significantly lower than those observed in plain scans [20]. Visceral fat in the arterial phase and the 3-minute delay phase were also found to be decreased by 20 cm^2^ on average relative to the plain scan phase, possibly due to the increased density of fat surrounding the intestines or due to contrast agent entry into visceral fat or peripheral microvessels, thus reducing the amount of adipose tissue meeting measurement threshold requirements [21]. As such, we analysed plain scan images in the present study. For these measurements, the contour of the middle portion of the abdominal wall muscle group was manually drawn on the scan images, and the adipose tissue area meeting the Hounsfield unit threshold (−150 to −50) was calculated automatically, as in prior reports [22]. The area between the outside of this contour and the skin was defined as the SFA, while the inner area of the contour was used to quantify the VFA. Two senior radiologists independently conducted all measurements. Total fat area (TFA) and rVFA were calculated as follows: TFA = VFA + SFA; rVFA = (VFA/TFA) × 100%.

### Statistical analysis

Continuous variables are given as means ± standard deviations, while categorical variables are expressed as numbers (percentages). Kolmogorov–Smirnovand Shapiro-Wilk tests were used to assess the normality of continuous variables when the number of patients being analysed was > 50 or < 50, respectively. Continuous data were then analysed via two sample Student’s t-tests when normally distributed with homogeneity of variance between groups, whereas they were otherwise analysed via Mann–Whitney U-tests. Categorical variables were compared via chi-squared tests. Correlations between age and continuous variables were evaluated, and a generalized linear model was then used to evaluate age-related variables among groups.

Univariate logistic regression analyses were used to examine relationships between T-stage and other ccRCC patient variables. Variables significant in initial univariate logistic regression analyses were incorporated into a subsequent multivariate logistic regression analysis, with the area under the ROC curve (AUC) being used to assess the predictive value of this model.

R v4.0.2 was used for all statistical testing, with a two-tailed *p* < 0.05 being the significance threshold.

## Results

### Clinical characteristics

**Figure 2.**
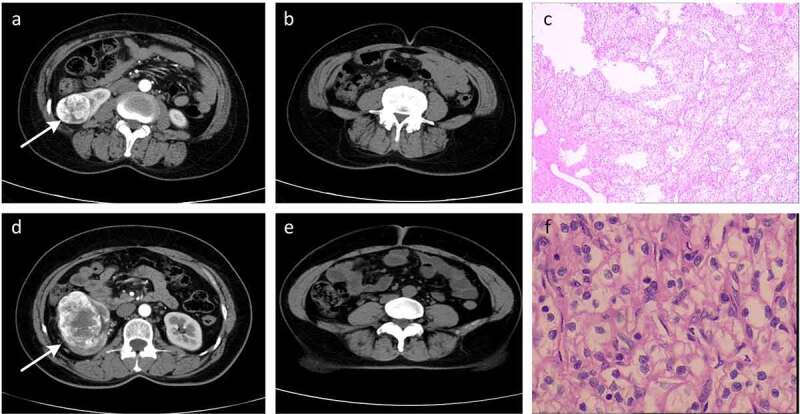
Representative radiographic and histological images of patients. (a-c) Low-T-stage ccRCC in a 49-year-old woman. (d-f) High-T-stage ccRCC in a 54-year-old woman. (a) Enhanced CT showing right renal tumour (white arrow). (b) CT image with a relative visceral fat area (rVFA) of 18.2%. (c) Histologic photomicrograph confirming that the tumour did not involve capsule so as to be T1 stage. (d) Enhanced CT showing right renal tumour (white arrow). (e) CT image with a relative visceral fat area (rVFA) of 35.0%. (f) Histological micrographs confirming that the tumour involved the capsule, but was confined to the perirenal fascia so as to be T3 stage

The baseline characteristics of patients enrolled in this study are shown in Table 1, with patients being stratified based upon disease T stage. In total, 253 patients (182 males and 71 females) from Yantai Yuhuangding hospital were included in this study. These patients had a mean age of 59.6 (± 10.8) years. Patients with high T-stage ccRCC exhibited a higher VFA (160.9 vs 136.7, *p* = 0.01) and rVFA (49.6 vs 41.1, *p*< 0.001), as well as a lower SFA (163.5 vs 195.0, *p* = 0.004) relative to those with low T-stage disease. Representative radiographic and histological images of patients in these two groups are shown in Figure 2.

**Table 1.** The baseline characteristics and fat measurements of patients stratified by T stage

Variable	Total (*n* = 253)	T12 (*n* = 178)	T34 (*n* = 75)	*p*	p*
Age, year	59.63 ± 10.75	57.97 (10.86)	63.56 (9.43)	**<0.001**^a^	
Sex, *n* (%) Female					
	71 (28.06)	59 (33.15)	12 (16.00)	**0.009**^b^	
Male	182 (71.94)	119 (66.85)	63 (84.00)		
BMI	25.50 ± 3.92	25.64 ± 3.83	24.38 ± 4.60	0.293^a^	0.441^d^
VFA, cm^2^	143.91 ± 64.32	136.74 ± 60.76	160.94 ± 69.57	**0.010**^a^	
SFA, cm^2^	185.70 ± 78.67	195.04 ± 79.88	163.53 ± 71.43	**0.004 **^c^	
TFA, cm^2^	329.61 ± 120.58	331.78 ± 116.52	324.47 ± 130.38	0.675^a^	
rVFA, %	43.61 ± 10.48	41.11 ± 10.51	49.55 ± 7.69	**<0.001 **^c^	**<0.001**^d^

*p*< 0.05 is indicated by boldface

^a^Student’s t-test; ^b^Chi-squared test; ^c^Mann–Whitney U test; ^d^General Linear Model; *adjusted p value

ccRCC: clear cell renal cell carcinoma; BMI: body mass index; VFA: visceral fat area; SFA: subcutaneous fat area; TFA: total fat area; rVFA: relative visceral fat area

### Analysis of the relationship between T-stage and visceral fat area in the overall patient population

**Figure 3.**
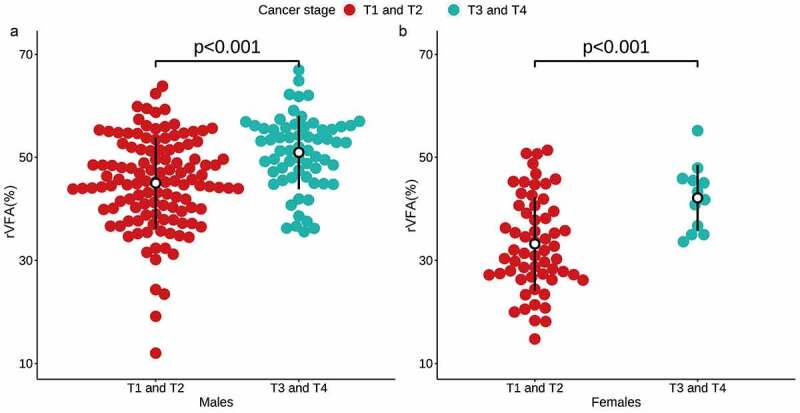
The relative visceral fat area (rVFA) was significantly different in both males (a) and females (b) based on T stage

Following adjustment for age, patients with high T-stage disease exhibited a higher rVFA relative to patients with low T-stage disease irrespective of sex (49.6 vs 41.1, *p*< 0.001) (Figure 3). However, VFA, SFA, and TFA did not differ significantly between these two patient groups (Table 1). In a univariate logistic regression analysis, rVFA (OR 1.103, 95% CIs 1.067–1.145, *p*< 0.001), age (OR 1.055, 95% CIs 1.026–1.087, *p*< 0.001), sex (OR 0.384, 95% CIs 0.185–0.746, *p* = 0.007), VFA (OR 1.006, 95% CIs 1.002–1.010, *p* = 0.007), and SFA (OR 0.994, 95% CIs 0.990–0.998, *p* = 0.004) were related to high T-stage ccRCC. In a subsequent multivariate analysis incorporating these same variables, rVFA (OR 1.154, 95% CIs 1.037–1.294, *p* = 0.011) and age (OR 1.050, 95% CIs 1.018–1.084, *p* = 0.002) were identified as significant predictors of high-T-stage ccRCC (Table 2).

**Table 2.** Univariate and multivariate models for predicting ccRCC T stage

	Univariate analysis		Multivariate analysis	
	OR (95% CI)	*p*	OR (95% CI)	*p*
Total (n = 253)				
Age, years	1.055 (1.026–1.087)	**<0.001**	1.050 (1.018–1.084)	**0.002**
Sex, female	0.384 (0.185–0.746)	**0.007**	0.946 (0.394–2.210)	0.900
BMI, kg/m2	0.915 (0.790–1.046)	0.214		
VFA, cm2	1.006 (1.002–1.010)	**0.007**	0.992 (0.976–1.008)	0.313
SFA, cm2	0.994 (0.990–0.998)	**0.004**	1.006 (0.991–1.020)	0.448
TFA, cm2	0.999 (0.997–1.002)	0.659		
rVFA, %	1.103 (1.067–1.145)	**<0.001**	1.154 (1.037–1.294)	**0.011**
Male (n = 182)				
Age, years	1.050 (1.019–1.085)	**0.002**	1.046 (1.013–1.083)	**0.007**
BMI, kg/m2	0.910 (0.774–1.053)	0.225		
VFA, cm2	1.004 (0.999–1.008)	0.118		
SFA, cm2	0.996 (0.991–1.001)	0.102		
TFA, cm2	1.000 (0.997–1.002)	0.975		
rVFA, %	1.100 (1.054–1.153)	**<0.001**	1.097 (1.050–1.151)	**<0.001**
Female (n = 71)				
Age, years	1.098 (1.019–1.207)	**0.028**	1.064 (0.979–1.178)	0.179
BMI, kg/m^2^	0.884 (0.513–1.268)	0.559		
VFA, cm^2^	1.015 (1.001–1.031)	**0.049**	1.000 (0.981–1.019)	0.968
SFA, cm^2^	0.994 (0.985–1.002)	0.166		
TFA, cm^2^	0.999 (0.993–1.005)	0.763		
rVFA, %	1.131 (1.045–1.245)	**0.005**	1.109 (1.008–1.239)	**0.045**

*p*< 0.05 is indicated by boldface

ccRCC: clear cell renal cell carcinoma; BMI: body mass index; VFA: visceral fat area; SFA: subcutaneous fat area; TFA: total fat area; rVFA: relative visceral fat area

### Sex-specific differences in the relationship between T-stage and abdominal fat area

The relationship between sex-specific abdominal fat parameters and ccRCC T-stage were next evaluated (Table 2). In univariate logistic regression analyses, rVFA (OR 1.100, 95% CIs 1.054–1.153, *p*< 0.001) and age (OR 1.050, 95% CIs 1.019–1.085, *p* = 0.002) were associated with high-T-stage ccRCC in males, while in females, rVFA (OR 1.131, 95% CIs 1.045–1.245, *p* = 0.005), age (OR 1.098, 95% CIs 1.019–1.207, *p* = 0.028), and VFA (OR 1.015, 95% CIs 1.001–1.031, *p* = 0.049) were related to high-T-stage ccRCC. Subsequent multivariate logistic regression analyses revealed rVFA (OR 1.097, 95% CIs 1.050–1.151, *p*< 0.001) and age (OR 1.046, 95% CIs 1.013–1.083, *p*＝0.007) to be predictors of high-T-stage ccRCC in males, while in females only rVFA (OR 1.109, 95% CIs 1.008–1.239, *p*＝0.045) was an independent predictor of advanced disease.

### Validation of the predictive value of rVFA

**Figure 4.**
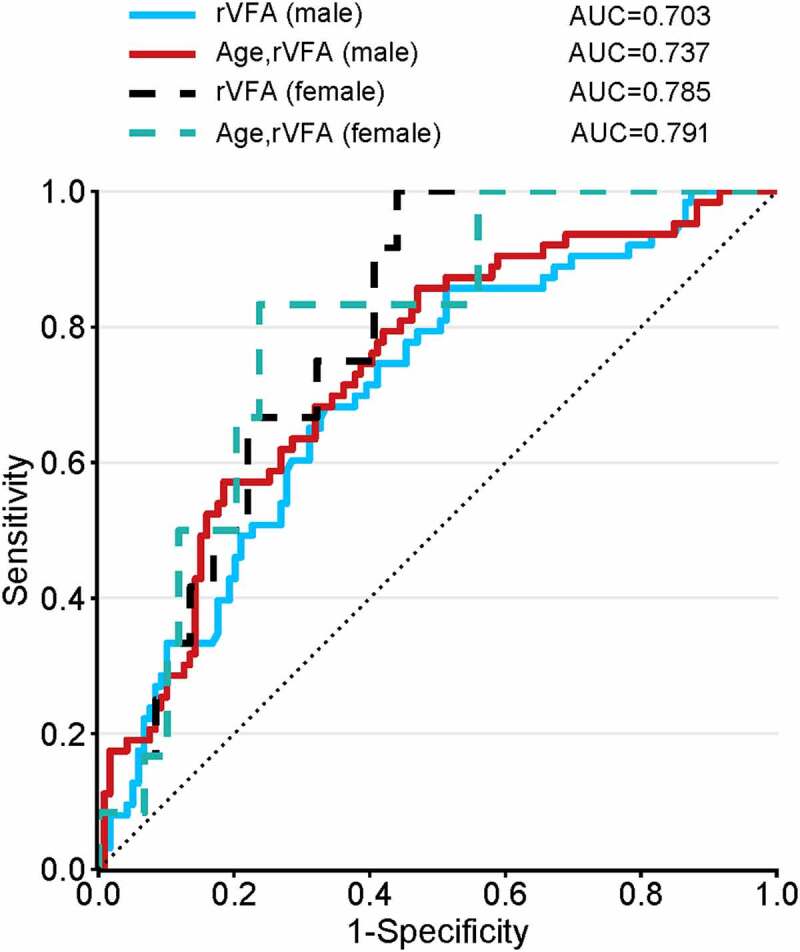
Comparisons of ROC curve analysis for univariate and multivariate models. Both the rVFA in males (blue solid line) and females (black dotted line) demonstrated good predictive power. In multivariate model incorporating age, good predictive power of rVFA was also observed irrespective of sex

An analysis conducted for males revealed rVFA to be an independent predictor of advanced disease in a univariate model (AUC = 0.703), while this AUC rose to 0.737 in a multivariate model incorporating age. Similarly, the rVFA AUC was an independent predictor of advanced disease in females in a univariate model (AUC =** **0.785), and in a multivariate model, the AUC of age and rVFA rose to 0.791 (Figure 4). As such, good predictive power was observed for all ccRCC patients irrespective of sex.

## Discussion

In addition to being the most common RCC subtype, ccRCC is closely associated with obesity [23]. This analysis indicated that rVFA was significantly higher in ccRCC patients with high-T-stage disease relative to those with low-T-stage disease in both males and females. Visceral fat composition is thus an independent predictor of ccRCC T staging irrespective of patient sex.

The International Diabetes Federation consensus statement indicates that waist circumference is a better metric for visceral obesity relative to BMI [24], but it is nonetheless a limited tool for analysing such obesity due to the cut-offs for defining obesity based on waist circumference vary according to ethnicity. As such, this organization also suggests that the measurement of VFA via MRI or CT scanning is more precise as a means of defining abdominal obesity. Otunctemur et al. determined that visceral fat is a significant predictor of tumour grade and size in ccRCC patients [25], and such correlations have also been confirmed for small RCC [26]. One multicenter study conducted in China revealed ccRCC patients’ visceral fat to be predictive of RCC pathological subtype [27]. As such, visceral fat can be used as a predictor of RCC development and progression. However, these prior studies did not specifically examine the relationship between rVFA and RCC disease parameters. T stage is an important metric used for the preoperative staging of RCC, guiding patient treatment and prognostic evaluation [28]. In this analysis, we used rVFA as a biomarker to predict ccRCC patient T stage while taking patient sex into account, as such an analysis has not been previously reported.

From a mechanistic perspective, obesity can drive oncogenesis through the decreased secretion of adiponectin and the chronic production of adipokines, insulin, insulin-like growth factor, inflammatory cytokines and tumour necrosis factor including IL-6 and TNFa. These factors can drive the proliferation of cancer cells and can promote angiogenesis, potentially facilitating tumour progression [29]. Conditioned medium derived from perirenal adipose tissue isolates from ccRCC patients significantly enhanced the migration of human RCC and ccRCC (ACHN and caki-2) cells through the adipose tissue-derived secretion of WNT-related factors that promote tumour progression [30]. Despite these results, the specific role of fat in RCC progression remains controversial [10,19,31–34]. Mizuno et al. determined that VFA is an independent predictor of metastatic RCC patient survival, improving the PFS and OS of these individuals [31]. Obesity-related survival advantages have also been reported in other studies [32], in what has been deemed an ‘obesity paradox’ [33,34]. The same has been found to be true in asian populations [10,19], with Lee et al. having found high visceral fat levels to be associated with longer specific survival in a Korean advanced RCC patient population [19]. Similarly, in one prognostic study of 295 Japanese patients with local RCC, researchers determined higher visceral fat levels to predict better recurrence-free survival [10]. The obesity paradox has been observed in different cancer settings and is not limited to non-metastatic disease. This phenomenon has been found in both elderly patients with acute myeloid leukaemia [35] and patients with colorectal metastases [36]. Memorial Sloan Kettering Cancer Center explained a potential genetic mechanism of ‘obesity paradox’. They found lower expression of the gene fatty acid synthase (FASN) in people who were obese; FASN encodes the enzyme fatty acid synthase that making fatty acids – an essential source of energy; the altered gene expression may have led to slower-growing kidney tumours [34]. However, other studies have detected no such relationship between survival and adiposity in western patients with non-metastatic ccRCC [34]. The role of visceral fat as an influencer of RCC progression is thus complex. In this study, we sought to explore this relationship by separating ccRCC patients according to sex and T-stage in an effort to mitigate sex-related differences in patient outcomes. We did not detect any correlation between SFA or VFA and ccRCC T stage, whereas rVFA was an independent predictor of T stage in both males and females. This is different from the findings of Hu et al. [13], who found rVFA to effectively predict high-grade ccRCC in female but not male patients. However, stage IV RCC patients were not included in their study, potentially explaining the differences between our results and their prior findings. Oestrogen levels can also influence fat distribution and functionality [12]. As oestrogen was not included in these analyses, this may have contributed to discrepancies in our results. This also suggests that visceral fat is not the sole factor influencing RCC progression, with such adiposity likely synergizing with factors including hormone levels, pathological type, stage, and treatment approach to influence patient outcomes. Future research will be required to test these relationships further and to expand on the present results.

There are multiple limitations to this study. For one, our sample size was limited, particularly for female patients owing to the lower rate of ccRCC among females [37]. Second, this was a single-centre retrospective study lacking any external data to validate our predictive model. Finally, we did not include sex hormones such as oestrogen as variables in our models, potentially constraining their predictive power.

In conclusion, visceral fat levels can be used to reliably predict ccRCC T stage, without any apparent difference in predictive efficacy when comparing males and females. However, further research is essential to understand the mechanisms whereby visceral fat can influence ccRCC development in order to better prevent and treat this deadly cancer type.
